# Development of a master–slave 3D printed robotic surgical finger with haptic feedback

**DOI:** 10.1007/s11701-024-01819-8

**Published:** 2024-01-18

**Authors:** Jamal T. Hamdi, Shadi Munshi, Sufyan Azam, Aiman Omer

**Affiliations:** 1 Surgical Department, Umm Al-Qura University, Makkah, Saudi Arabia; 2 Mechanical Engineering Department, Umm Al-Qura University, Makkah, Saudi Arabia; 3 School of Science and Engineering, Waseda University, Tokyo, Japan

**Keywords:** Laparoscopic, Robotic surgery, 3D printing, Master–slave, Robotic surgical finger, Haptic feedback

## Abstract

Robotic surgery started nearly 30 years ago. It has achieved telepresence and the performance of repetitive, precise, and accurate tasks. The “master–slave” robotic system allows control of manipulators by surgeon at distant site. Robotic surgical fingers were developed to allow surgeons to move them with accuracy through sensors fixed on surgeon’s hand. Also, haptic sensors were developed to allow transmission of sensation from robotic finger to surgeon’s finger. A complete system of a, 3D printed by a stereolithography (SLA) 3D printer, robotic surgical finger with haptic feedback system is proposed. The developed system includes a master glove that controls the motion of a 3DOF robotic slave finger while getting haptic feedback of force/pressure exerted on it. The precise control of the slave robotic finger was achieved by applying a Proportional Integral and Derivative (PID), fast and robust, control algorithm using an Arduino based hardware and software module. The individual joint angles, metacarpophalangeal joint (MCP) and proximal interphalangeal joint (PIP), and wrist were measured using rotatory and inertial sensors respectively. The degree of movement for MCP, PIP, and Wrist joints were measured to be 0–86°, 0–71°, and 0–89° respectively. Motion to the robotic finger is mimicked by a glove motion requiring minimal learning curve for the device. The collected data for the slave motion is in good agreement with the master-glove motion data. The vibro-tactile haptic feedback system was developed to distinguish between three different materials to mimic human flesh, tumor, and bone. The master–slave system using robotic surgical finger with good simultaneous movement to surgeon’s finger and good haptic sensation will provide the surgeon with the opportunity to perform finger dissection in laparoscopic and robotic surgery, as it used to be in open surgery. 3D bio printing will make this process even cheaper with the added advantage of making surgical tools locally according to the need of the surgery. An ongoing work is to develop silicone based 8 mm robotic surgical finger with multiple type haptic feedback.

## Background

The application of robotics in medicine started nearly 30 years ago. The use of robots in medical procedure started from the need to accomplish two objectives: telepresence and the performance of repetitive, precise, and accurate tasks. The “master–slave” robotic system with remote manipulators operated by a surgeon at a workstation was developed in the early 1990s. Automated medical procedure or robotic surgery has the benefits of 3D vision, steady and magnified imaging, Endo-Wrist instruments, physiologic tremor filtering, and motion scaling over laparoscopic surgery; however, it lacks in force and tactile feedback. In general surgery, the oncological, perioperative, and functional outcomes of robotic and laparoscopic surgical procedures are similar; however greater expenses and absence of haptic feedback are the major obstacles of current robotic technology to turn into the standard benchmark of minimally invasive surgery (MIS) around the world. Until now, the surgical time and hospital costs have been invariably unfavorable to robotics in cost-effectiveness studies published to date. Therefore, development in robotic surgery should involve cost reduction and the development of new technologies [1, 2]. The da Vinci robot is a master–slave device developed by Intuitive Surgical Inc. It is currently the widest spread robotic surgical system [3]. Four generations of the da Vinci system have been introduced over the last 2 decades [4]. Robotic surgery is reserved for major surgeries and particularly for those in the pelvis and posterior abdominal cavity. Surgery can be performed through a single port or multiports [5]. Miniature in vivo robots have the potential to address the limitations of using articulated instrumentation to perform advanced laparoscopic surgical procedures. Once inserted into the peritoneal cavity, the robot provides a stable platform for visualization with sufficient dexterity and speed to perform surgical tasks from multiple orientations and workspaces [6]. There were attempts to produce Robotic surgical hands, but those were modifications of the da Vinci system and still expensive [7]. 3D printing is mainly used to create models to help surgeons plan for surgery [8]. There are plans to be used in space missions [9] and advocated to produce surgical instruments at a very small cost comparing with stainless instruments [10, 11].

We report a robotic surgical finger developed in Umm Al-Qura University, Makkah, Saudi Arabia by collaboration between staff in the Surgical Department and Mechatronics section of the Mechanical Engineering department. This paper is based on US patent from the group [12]. This is a tool, when fully developed should have the potential of introduction through laparoscopic port in single port or multiport surgery and controlled by gloves worn a surgeon at a distant site. The surgeon can be in the same room, but also he can be on the other side of the globe using 5G Telecommunication Technology [13]. The robotic finger will have a haptic sensation to transmit sensation, pressure, and other modalities to the Surgeon’s finger. Such applications should lay the base for a new type of surgery which will combine laparoscopic and robotic surgery. We coin the phrase (laparobotic surgery) for this type of surgery, as it includes (laparo) from laparoscopic, and (robotic) to reflect robotic surgery. This is developed locally using a 3D printer with low cost and high versatility and adaptation. The model can be adjusted according to the need for the surgery and can be used multiple times because its introduction into the abdominal cavity is through the protective sterile covering.

## Design methodology

**Fig. 1:**
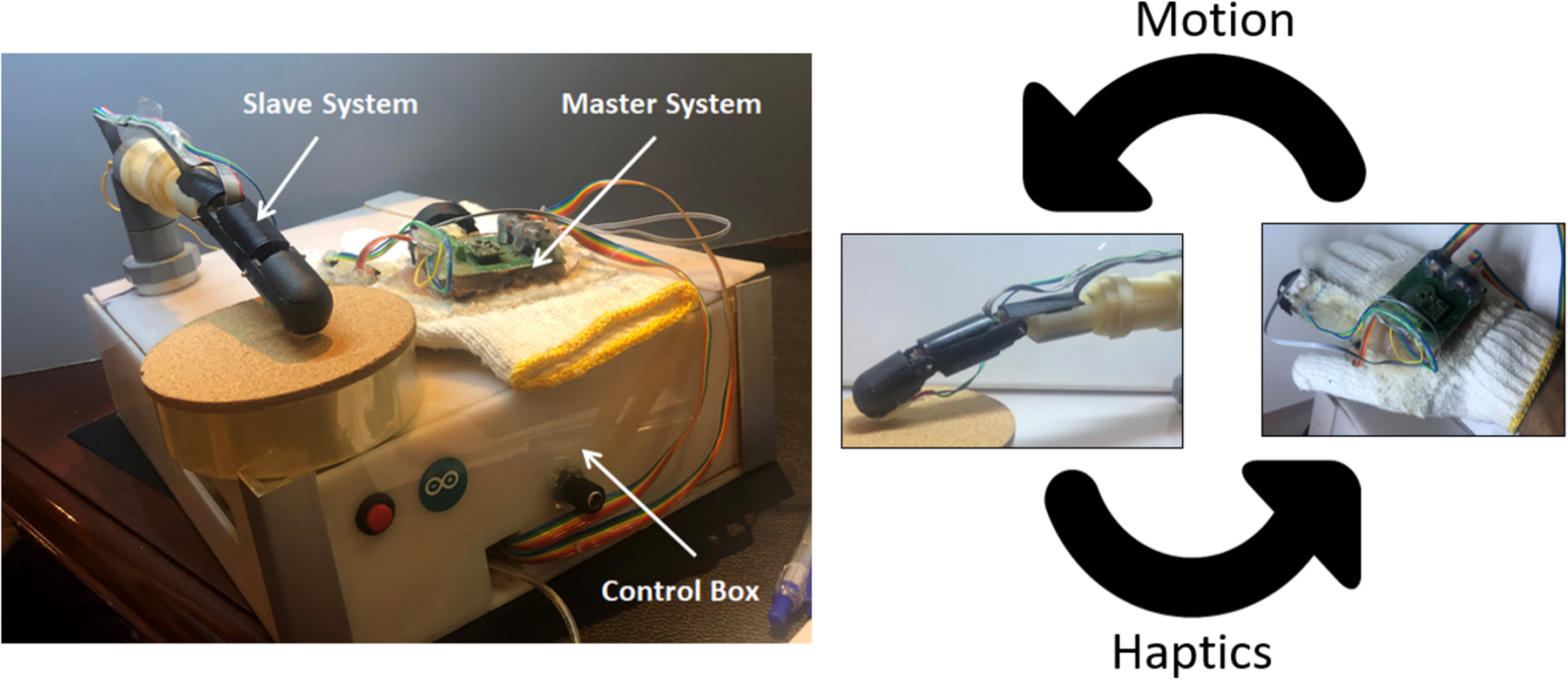
Master glove and robotic slave systems

The proposed robotic surgical finger design consists of a master system, a slave system, and a controller box as shown in Fig. 1.

## Master system design

**Fig. 2:**
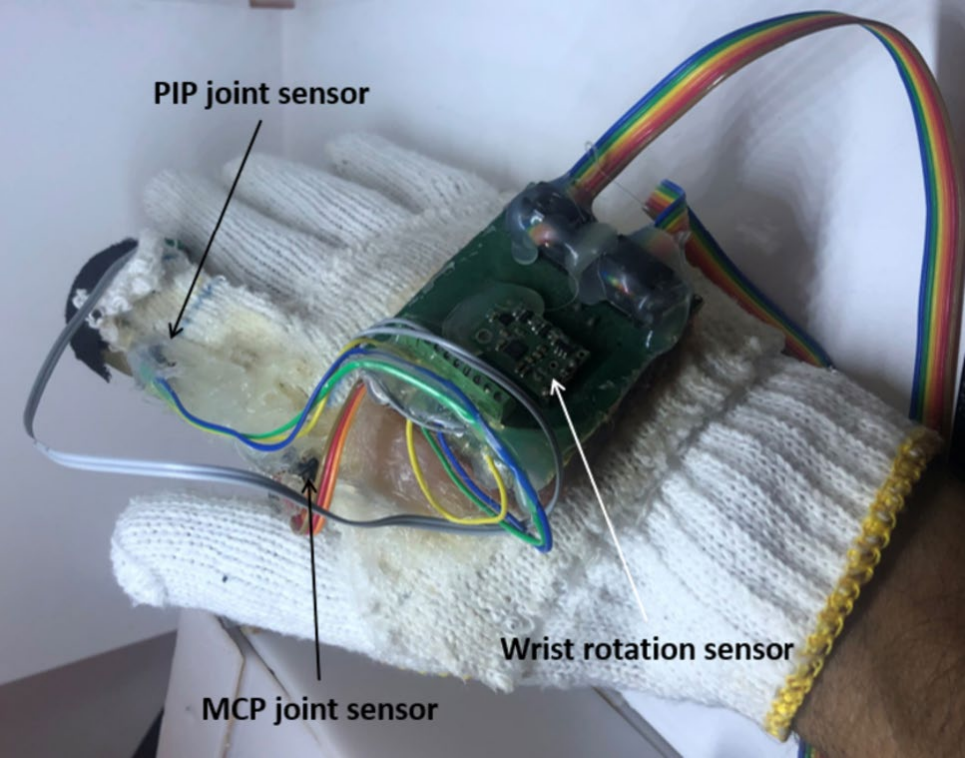
Sensing system in the master glove

The master system consists of a wearable glove equipped with three sensors that can accurately sense human finger movements and an electric circuit to transfer master finger position information to the controller. The controller then processes this information and sends signals to the salve to accurately control the movement of the 3D printed surgical finger as shown in Fig. 2.

The sensors used in the master glove are rotary encoders for measuring two individual joint angles, metacarpophalangeal joint (MCP) and proximal interphalangeal joint (PIP), and a Mini IMU-9 v5 inertial sensor [14] for the wrist rotation angle. To have haptic feedback, the master glove uses a vibro-tactile actuator.

## Slave system design

**Fig. 3:**
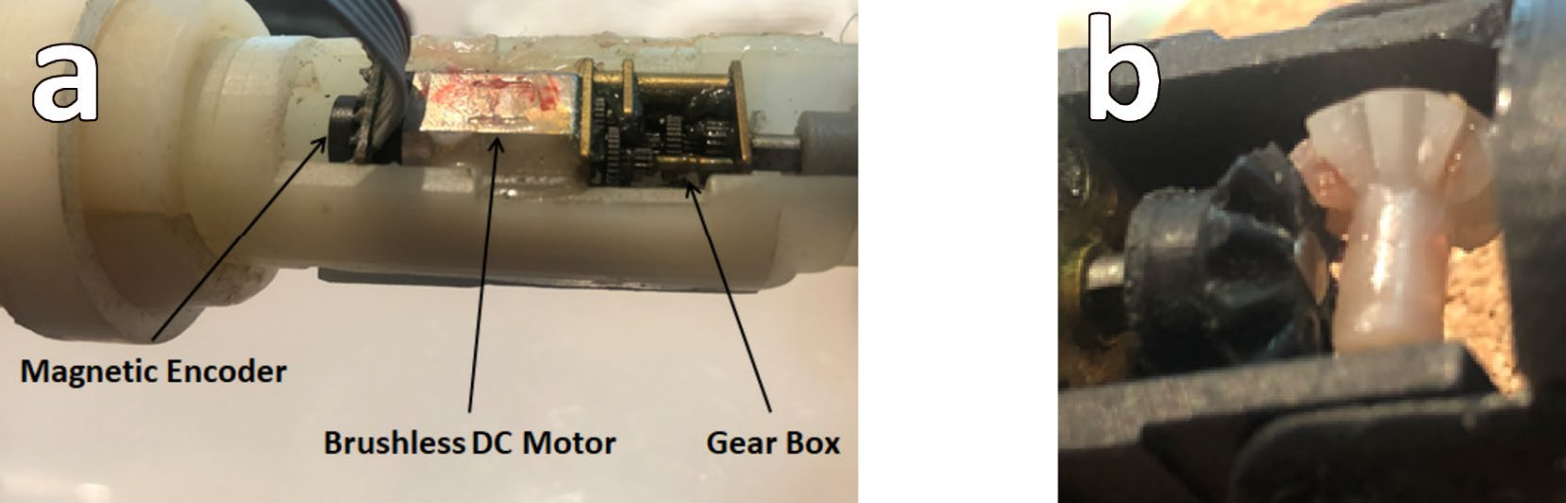
(**a**) Robotic finger joint actuator; (**b**) 3D printed mitre bevel gears

The slave system mainly consists of a 3D printed robotic surgical finger. The robotic finger is designed using SolidWorks to mimic a human’s finger with three degrees of freedom. It has 1 Degree of freedom (DOF) for the metacarpophalangeal (MCP) joint and 1DOF for the proximal interphalangeal joint (PIP). The distal interphalangeal joint (DIP) has a small angle for motion and is neglected in the design. The rotation of the wrist joint is 1DOF. Thus, the robotic finger has three degrees of freedom. Each degree of freedom is controlled using a micro-metal gear motor with a magnetic encoder for accurate tracking of the finger joints. The gear motor is a miniature high-power, 330 rpm, and 1.6 kg cm extrapolated stalling torque, brushed DC motor with 100:1 metal gearbox. The cross-section of the motor is 10 × 12 mm, and the gearbox output shaft is 9 mm long and 3 mm in diameter. The motor also has a 4.5 × 1 mm extended back shaft for magnetic position feedback encoder. The magnetic encoder uses Hall effect sensors and a magnetic disc to provide 12 counts per revolution (CPR) of the motor shaft as shown in Fig. 3a. A pair of miniature mitre bevel gears, with pitch circle diameter of 8 mm, teeth ratio 1:1, and having 14 number of teeth, were designed and manufactured to give angular motion to the robotic finger joints as shown in Fig. 3b.

**Fig. 4:**
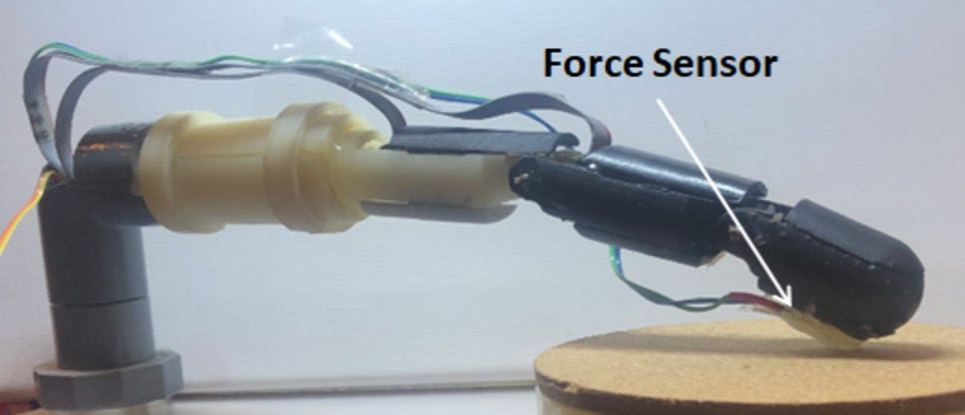
Robotic finger with a force sensor

The robotic finger is also equipped with a force sensor attached to the tip of the finger to provide actuation for haptic feedback (Fig. 4).

## Master slave control algorithm

**Fig. 5:**
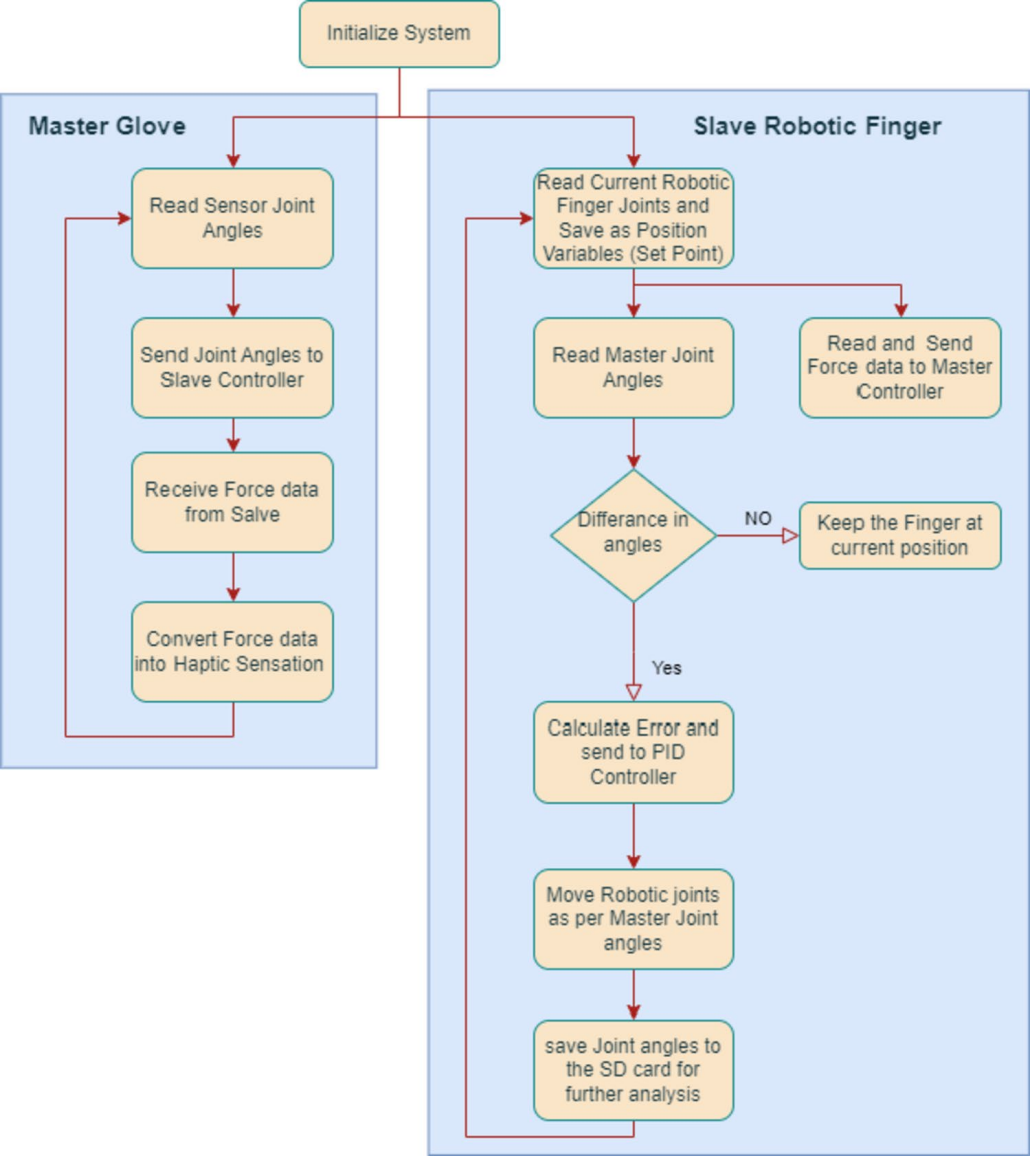
Flow chart of master slave algorithm

The precise control of the robotic finger is achieved by applying a Proportional Integral and Derivative (PID), fast and robust, control algorithm using a developed hardware and software module. Figure 5 shows master–slave control algorithm in terms of a flow chart.

**Fig. 6:**
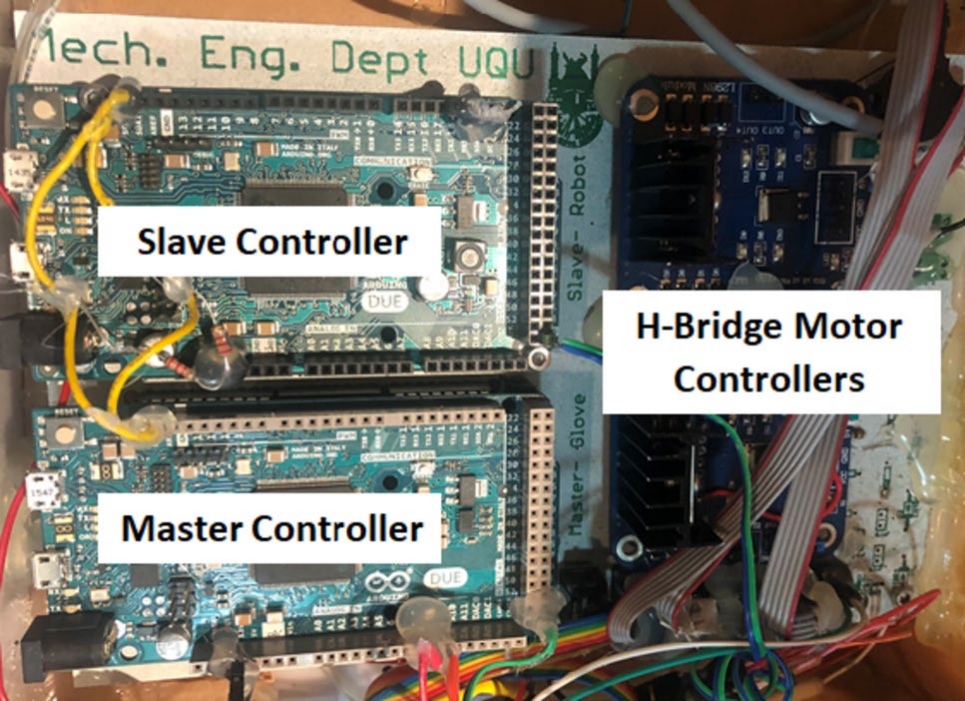
Hardware module for the complete system

Figure 6 shows the hardware which consists of two Arduino Due microcontrollers to sense master-glove angles and control robotic finger (slave) motion. Two dual-channel H bridges are used to move DC motors. The main electronic board was designed and manufactured using LPKF S103 printed circuit board plotter. The range of movement for the PIP joint is from 0 to 71°, and for MCP from 0 to 86°. Both movements are in the vertical plane to the finger axis. The wrist joint has a range of movement from 0 to 89° in rotational movement clockwise.

## Haptic system

**Fig. 7:**
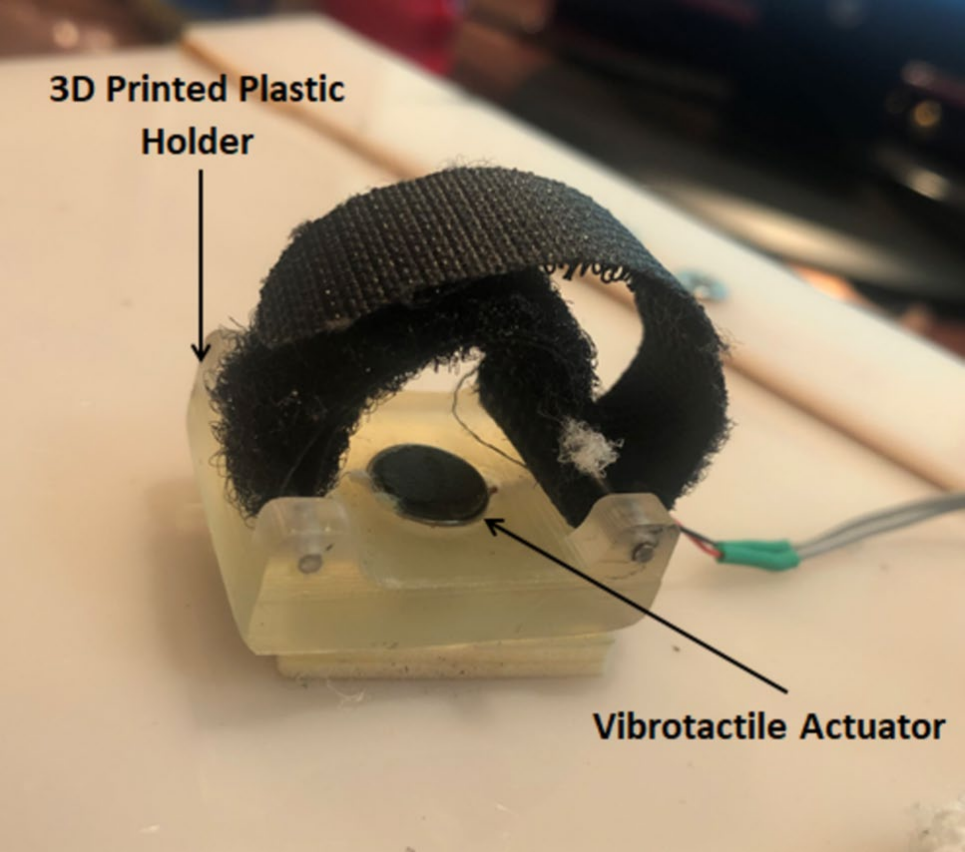
Shaftless vibro-tactile actuator

The proposed haptic system consists of a vibro-tactile feedback actuator and a force sensor (FSR), from Tekscan, mounted on the bottom tip of the robotic surgical finger. It is a piezoresistive force sensor having a sensing area of 3.8 mm in diameter and a response time less than 5 μs with standard force range of 18N. Vibro-tactile feedback is provided with a shaftless eccentric mass motor driven by a linear amplifier circuit. The actuator is 10 mm in diameter and is mounted to a wearable 3D printed plastic cap as shown in Fig. 7.

**Fig. 8:**
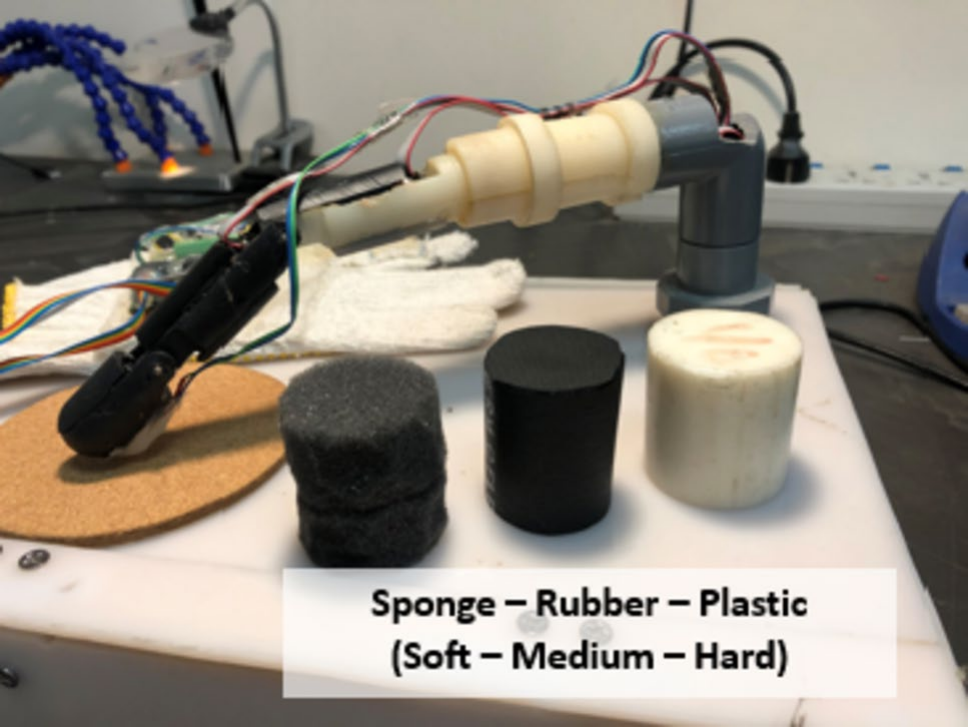
Testing surface levels

Three surface levels i.e., soft, firm and hard, were tested to mimic human flesh, tumor and bone as shown in Fig. 8.

**Fig. 9:**
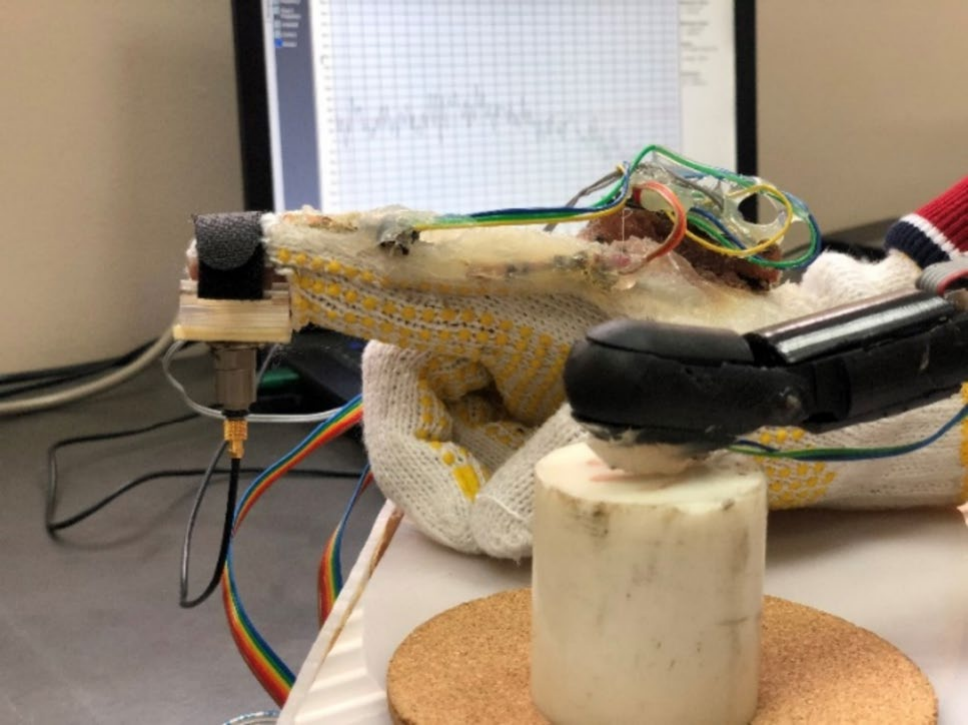
Vibration sensor for haptic feedback validation

When the robotic finger touches any surface, the pressure sensor mounted on it, sends a signal to the controller and the controller then transfers this signal to the actuator mounted on the master glove. This gives a feeling of haptic feedback to the person wearing the master glove. The haptic sensation for all three materials was validated using a vibration sensor at the tip of the master glove as in Fig. 9.

## Results

### Master–slave motion

**Fig. 10:**
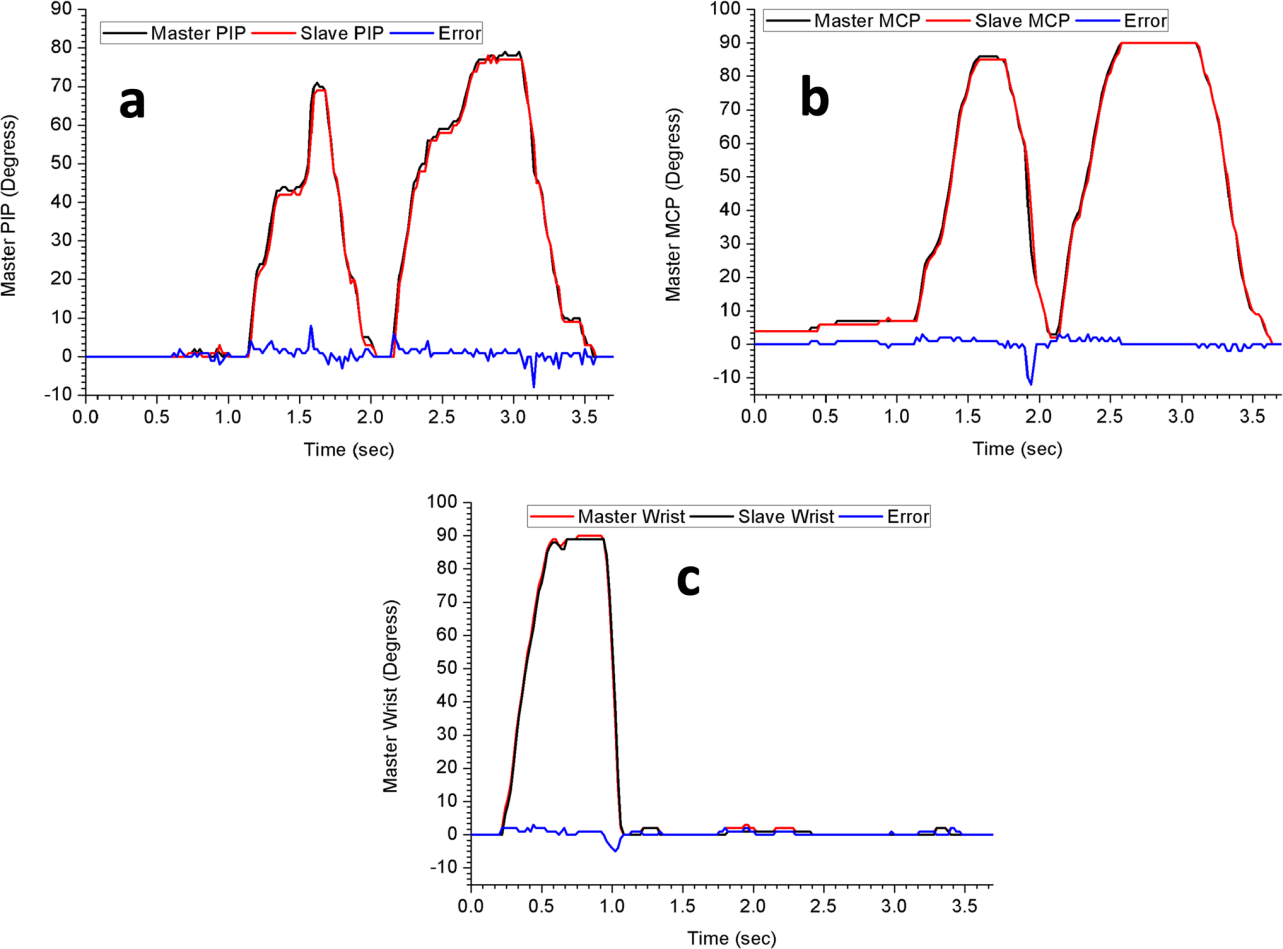
(**a**) PIP master and slave joint motion; (**b**) MCP master and slave joint motion; (**c**) Wrist master and slave joint motion

The developed system was tested for master and slave motion using the master glove and the slave robotic finger. The motion from the master finger was transferred to the robotic finger in real-time with high accuracy. The time delays for PIP, MCP, and wrist joints were 14, 6, and 11 ms (millisecond) respectively. The delay time depends on the speed of the master-glove operator. This was measured by plotting motion data of each joint on OriginPro 2018 (Data analysis software). The accuracy of the movement for all the three joints was measured to be within 1degree at slow movements. There was a 1degree lag between the slave PIP, MCP, and wrist joints as measured by plotting angles against time. The collected data for the slave motion is in good agreement with the master-glove motion data. Figure 10a and b show two complete master–slave motions of the PIP and MCP joints while Fig. 10c displays one complete movement of master and slave wrist joints. The degree of movement for MCP, PIP and Wrist joints were measured to be 0–86°, 0–71°, and 0–89° respectively. A slight rise in error in Fig. 10a is due to the transition from slow to fast movement from 3.5 to 21 rad/s. The same behavior can be observed in Fig. 10b and c.

### Master–slave haptic feedback

**Fig. 11:**
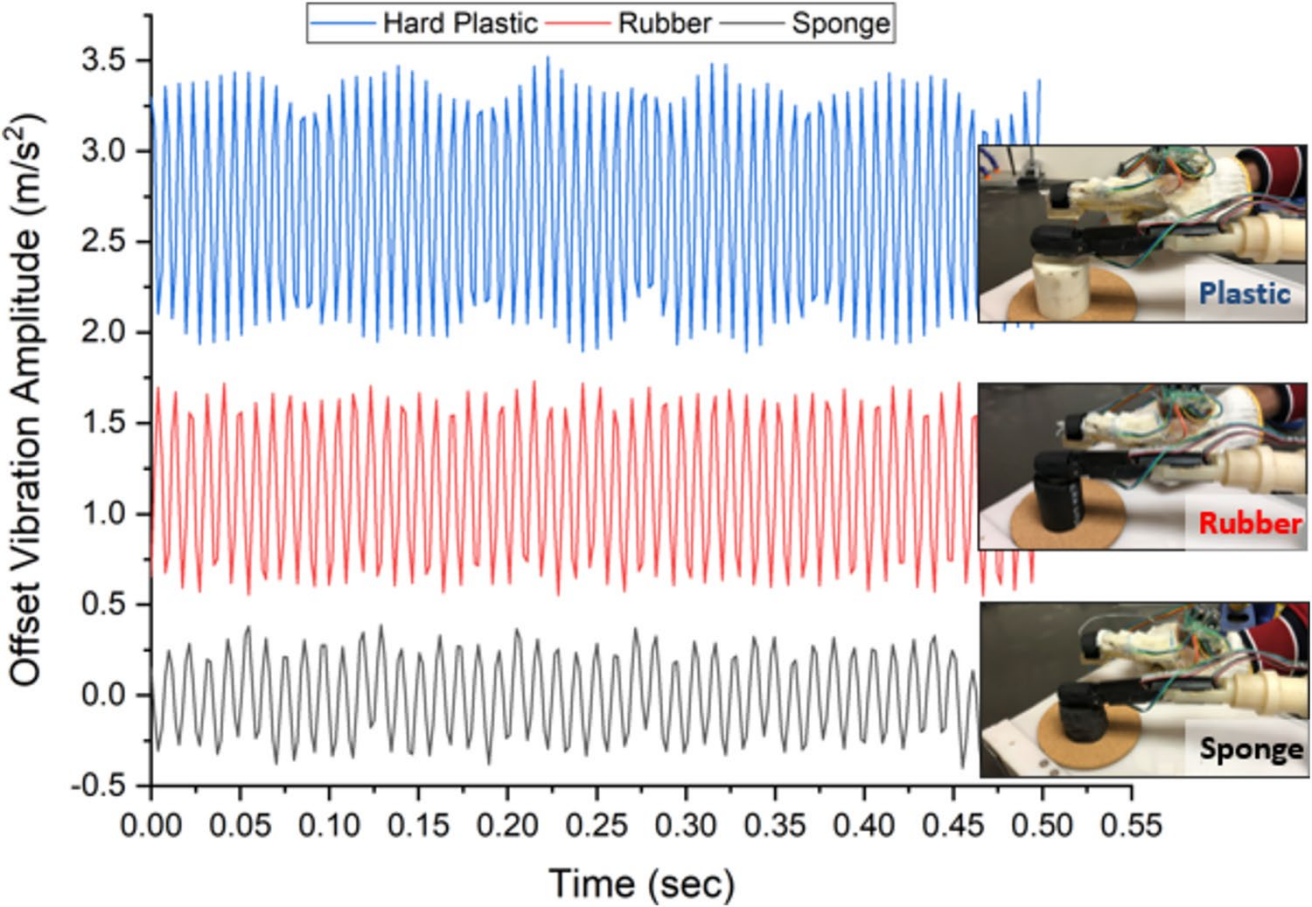
Vibro-tactile feedback response for different materials

Figure 11 shows the response of the vibro-tactile feedback recorded by the vibration sensor for the three materials. The Plastic (hard material) that mimics the human bone shows large amplitude of sensation as compared to the sponge (soft material) at the same joint movements.

## Discussion

A master–slave 3D printed robotic system was developed. Our robotic surgical finger has three joints. Each of the MCP and PIP joints has one DOF in flexion–extension plane with range of movement (ROM) of nearly 90° in each joint. The wrist joint has one DOF in rotational movement with a range of movement of nearly 90°. The master–slave joint movements showed exceptionally good correlation between the gloved surgeon’s finger and the robotic finger especially if rapid movements are avoided. This will restore to the surgeon the ability of finger dissection which he enjoyed in open surgery and lost in laparoscopic and robotic surgery. We used vibratory haptic sensors to transmit force sensation from the tip of robotic finger to the tip of the gloved surgeon’s finger. The amplitude of the vibration gives a good sensation to the surgeon to distinguish between hard, firm, and soft sensation. The collected data for the haptic feedback clearly distinguished between the three varied materials. The robotic surgical finger has a diameter of nearly 15 mm, and due to the limitations of 3D printing, we were not able to develop a small end effector of 10 mm size. Ongoing future work focuses on the reduction in the size because of the availability of micro servo motors of 6 mm in diameter and miniature metallic gears, which will enable surgeons to introduce it from 10 mm port. In its present size, it can be used in single port minimally invasive surgery. This will enable the surgeon to do effective safe finger dissection from distant sites, which he was able to do only from close range in open surgery.

Our project combines the development of robotic surgical finger with good haptic sensation, which can be controlled accurately by an endoskeleton system mounted into the surgeon’s glove. This finger is also personalized as it can be produced locally and cheaply with 3D printers according to need. The proof-of-concept surgical robotic finger developed here will form the basis of an innovation of surgery which will bring the major advantage of finger and hand dissection in open surgery to laparoscopic and Robotic surgery. The utilization of 3D printers will enable hospitals to produce cheap surgical fingers and tools that can be produced locally according to surgical needs and can be of single use or multiple uses.

The majority of the ongoing research concentrate on the production of 3D printed surgical instruments with [15, 16] or without haptic sensation for use in classical robotic surgery [16, 17]. Even some robotic hands like MUSHA Hand or Chinese hand were not intended as human hands, but as modification of surgical instruments [18, 19].

Our project combines robotic surgical finger with haptic sensation and 3D printing with vision of use in finger dissection in MIS (minimally invasive surgery) as it used to be in open surgery. This finger is controlled by endoskeleton wearable glove by surgeon. This finger can be developed later to three fingers hand for full manipulation of internal organs.

## Conclusion

Our robotic surgical finger with good simultaneous movement to surgeon’s finger and good haptic sensation will provide the surgeon with the opportunity to perform finger dissection in laparoscopic and robotic surgery. The restoration of the ability of the surgeon to use his hand as in open surgery should improve the quality and speed of surgery. We are currently developing three and five fingers robotic surgical hand to introduce hand dissection and hand mobilization of internal organs into laparobotic surgery. Also, we are testing different haptic modalities like pneumatic, thermal, and electrical to test their feasibility and efficiency.

The majority of research papers in this field are from engineers with a pure engineering point of view. Our research group is based on full collaboration between surgeons and engineers which start from assessing the need, building the concept, designing the innovation, production, modification, testing and then writing.

The application of master–slave technology in robotic surgery will add dexterity and haptic sensation to tele-surgery, which in combination with 5G technology in telecommunication will allow distant surgery to be performed safely. Robotic tele-surgery can provide intense and immediate health care services and can bring excellent opportunities to serve highly specialized skills worldwide [20]. The expansion of 5G technology has enabled some countries to conduct remote surgical procedures, tele-mentored and real-time interactive procedures on animal models, cadavers, and humans, demonstrating that 5G networks could offer a potential solution to previously experienced latency and reliability hurdles during the remote surgeries [13, 21].

In situ AI-empowered 3D-printing approaches could be integrated in future 3D-printing of functional materials and personalized biomedical devices [22]. The ongoing work is focused to develop an Artificial intelligence (AI) based master slave system made of 8 mm 3D printed silicone robotic surgical finger with multiple type haptic feedback, that can later provide a base for the development of a laparobotic surgical devices. The addition of AI to laparobotic surgery will take us a step forward to fully automated surgery in future.

## Data Availability

The datasets used and/or analysed during the current study are available from the corresponding author on reasonable request.

## Abbreviations

3D: Three dimensions
PIP: Proximal interphalangeal joint
MCP: Metacarpophalangeal joint
DIP: Distal interphalangeal joint
DOF: Degree of freedom
AI: Artificial intelligence
5G: The 5th generation of mobile network
MIS: Minimally invasive surgery
